# Systemic Candida albicans Infection in Mice Causes Endogenous Endophthalmitis via Breaching the Outer Blood-Retinal Barrier

**DOI:** 10.1128/spectrum.01658-22

**Published:** 2022-08-01

**Authors:** Sneha Singh, Sukhvinder Singh, Ashok Kumar

**Affiliations:** a Department of Ophthalmology, Visual and Anatomical Sciences/Kresge Eye Institute, Wayne State University School of Medicine, Detroit, Michigan, USA; b Department of Biochemistry, Microbiology, and Immunology, Wayne State University School of Medicine, Detroit, Michigan, USA; University of Iowa Hospitals and Clinics

**Keywords:** *Candida albicans*, endogenous endophthalmitis, fungal endophthalmitis, inflammation, retina, eye, blood-retinal barrier, endophthalmitis, innate immunity

## Abstract

Candida albicans is the leading cause of endogenous fungal endophthalmitis; however, its pathobiology studies are limited. Moreover, the contribution of host factors in the pathogenesis of *Candida* endophthalmitis remains unclear. In the present study, we developed a murine model of C. albicans endogenous endophthalmitis and investigated the molecular pathobiology of ocular candidiasis and blood-retinal barrier permeability. Our data show that intravenous injection of C. albicans in immunocompetent C57BL/6 mice led to endogenous endophthalmitis without causing mortality, and C. albicans was detected in the eyes at 3 days postinfection and persisted for up to 10 days. The intraocular presence of C. albicans coincided with a decrease in retinal function and increased expression of inflammatory mediators (tumor necrosis factor alpha [TNF-α], interleukin 1β [IL-1β], MIP2, and KC) and antimicrobial peptides (human β-defensins [hBDs] and LL37) in mouse retinal tissue. C. albicans infection disrupted the blood-retinal barrier (BRB) by decreasing the expression of tight junction (ZO-1) and adherens junction (E-cadherin, N/R-cadherin) proteins. *In vitro* studies using human retinal pigment epithelial (ARPE-19) cells showed time-dependent activation of eIF2α, extracellular signal-related kinase (ERK), and NF-κB signaling and decreased activity of AMP-activated protein kinase (AMPK) leading to the induction of an inflammatory response upon C. albicans infection. Moreover, C. albicans-infected cells exhibited increased cellular permeability coinciding with a reduction in cellular junction proteins. Overall, our study provides new insight into the molecular pathogenesis of C. albicans endogenous endophthalmitis. Furthermore, the experimental models developed in the study can be used to identify newer therapeutic targets or test the efficacy of drugs to treat and prevent fungal endophthalmitis.

**IMPORTANCE** Patients with candidemia often experience endophthalmitis, a blinding infectious eye disease. However, the pathogenesis of *Candida* endophthalmitis is not well understood. Here, using *in vivo* and *in vitro* experimental models, we describe events leading to the invasion of *Candida* into the eye. We show that *Candida* from the systemic circulation disrupts the protective blood-retinal barrier and causes endogenous endophthalmitis. Our study highlights an important role of retinal pigment epithelial cells in evoking innate inflammatory and antimicrobial responses toward C. albicans infection. This study allows a better understanding of the pathobiology of fungal endophthalmitis, which can lead to the discovery of novel therapeutic targets to treat ocular fungal infections.

## INTRODUCTION

Infectious endophthalmitis is a vision-threatening eye infection (1–3). Among various pathogens, fungi are the second leading cause of exogenous and endogenous endophthalmitis after bacteria, wherein the microbe gains access to the ocular tissue via an external source of contamination or the hematogenous route, respectively (4, 5). *Candida* is the most common causative agent of endogenous fungal endophthalmitis in hospitals, with C. albicans being the most prevalent species (6–10). C. albicans is a commensal pathogen inhabiting the oral, gastrointestinal, and genital tracts, which, upon disruption of the protective barrier or alteration of the host immune response, can enter the bloodstream and cause infection (11). In addition to systemic infections, C. albicans can cause ocular candidiasis in approximately 10% to 25% of candidemia patients (1, 7, 12, 13). The *Candida* endogenous endophthalmitis is mainly associated with immunocompromised or immunosuppression conditions; however, C. albicans can also cause endophthalmitis in immunocompetent individuals (14–16).

Most studies related to *Candida* endophthalmitis are classified as clinical and epidemiological, where the focus of the study is to describe the risk factors (parenteral nutrition; immunosuppression; intravenous drug use; recent central venous catheters, including peripherally inserted central catheters; race; and age) involved in the pathogenesis (1, 7, 12, 13, 17). Surprisingly, the few experimental studies available (13, 18–21) have an emphasis on evaluating the efficacy of antifungal drugs and comparing the infectivity of different *Candida* species. Previously, we have established a murine model of Aspergillus and *Candida* endophthalmitis to characterize the retinal innate immune response and its pathogenesis (4, 22). However, in these studies, fungi were administered via intravitreal injections, representing the exogenous endophthalmitis models.

C. albicans infection was significantly associated with ocular candidiasis due to its enhanced capacity for tissue invasion, inflammation, and recruitment of neutrophils (4, 13). However, it is important to note that not every candidemia patient develops endophthalmitis. This is because the eye is protected from inflammatory cells and pathogen invasion by the blood-retinal barrier (BRB), composed of retinal pigment epithelium (outer BRB) and retinal endothelium (inner BRB) (23). The tight and adherens junction protein binds the BRB intracellular junctions to maintain the barrier integrity (24). Dysfunction or degradation of these junction proteins can lead to a breach of the BRB and contribute to endogenous endophthalmitis and loss of retinal function. Currently, the interactions of *Candida* with BRB cells remain elusive, as suitable experimental models are needed to study the pathogenesis of endogenous *Candida* endophthalmitis. We postulate that intracellular junction disruption and BRB permeability precedes C. albicans endogenous endophthalmitis.

Here, we sought to develop a murine model of endogenous C. albicans endophthalmitis using systemic routes of infection along with the characterization of its pathogenesis. The *in vivo* study was complemented with an *in vitro* model of C. albicans infection of human retinal pigment epithelial cells, constituting the outer BRB. Hence, the development of appropriate experimental models and an in-depth understanding of pathological changes during C. albicans endophthalmitis can lead to the identification of novel therapeutics to prevent fungal endophthalmitis.

## RESULTS

### Systemic *Candida* infection results in endogenous endophthalmitis in mice.

**FIG 1:**
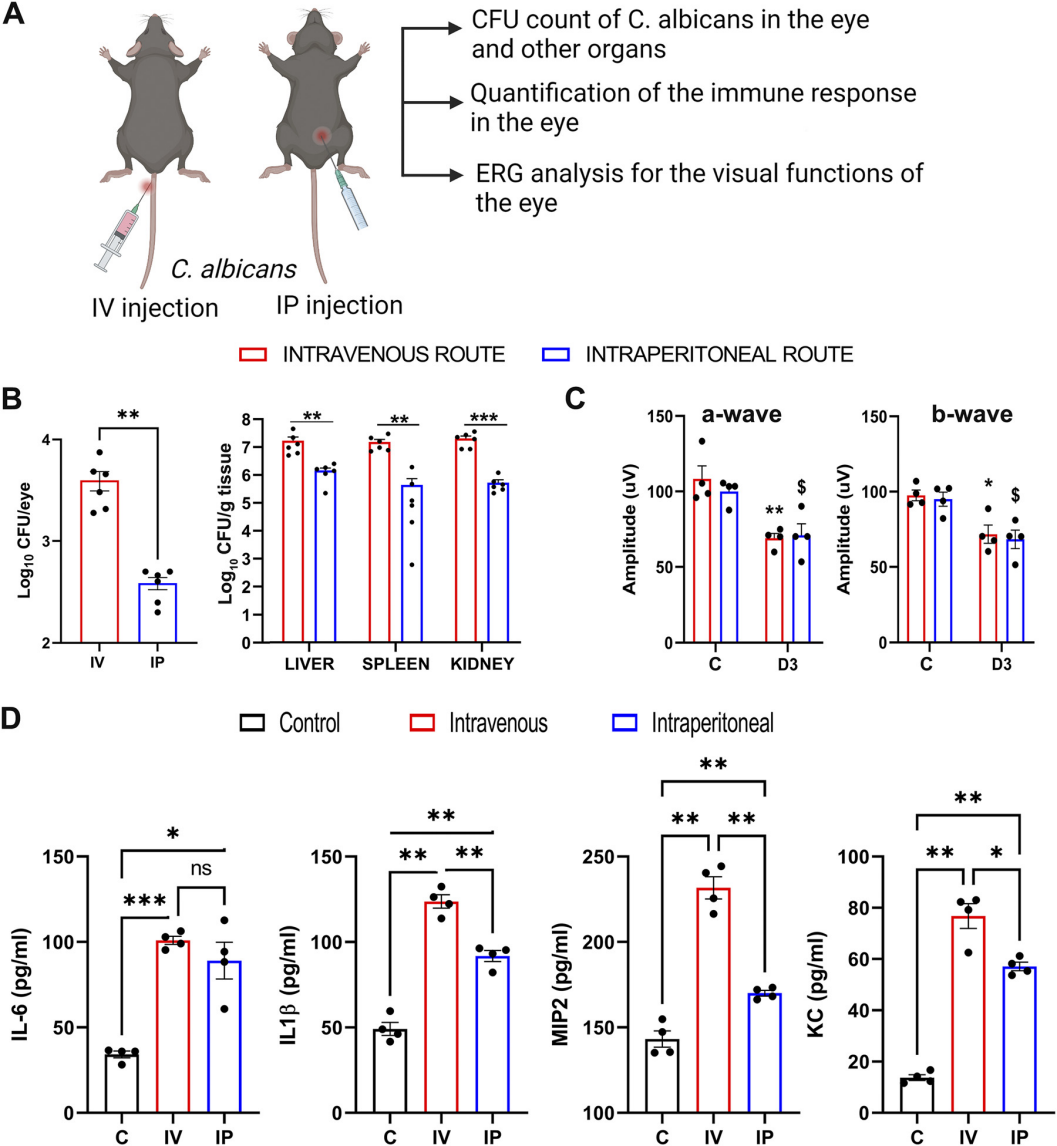
Systemic C. albicans infection causes endophthalmitis in C57BL/6 mice. (A) Schematic representation of the experimental setup showing intravenous or intraperitoneal injection of ~5 × 10^5^ CFU of Candida albicans in C57BL/6 mice (*n* = 4 to 6). (B) The eyes and indicated organs were harvested at 3 days (D3) postinfection, and fungal burden was estimated by the standard plate count method. (C and D) In another experiment, at D3, ERG response was measured in anesthetized mice (*n* = 4) (C), and enucleated whole-eye lysates were used to assess indicated inflammatory mediators by ELISA (D) and expressed as picogram per milliliter. ns, nonsignificant; *, *P* < 0.05; **, *P* < 0.01; ***, *P* < 0.001. Unpaired *t* test was used in panel B, one-way analysis of variance (ANOVA) and Šídák’s multiple-comparison test in panel C, and Dunnett’s multiple-comparison test in panel D. *, control versus i.v.; $, control versus i.p. (C).

Endogenous endophthalmitis is caused by the hematogenous spread of C. albicans in candidemia patients (14–16). To mimic the clinical scenario of systemic infection, we injected C. albicans into immunocompetent C57BL/6 (B6) mice via intravenous (i.v.) and intraperitoneal (i.p.) routes and assessed intraocular fungal burden, electroretinography (ERG) response, and inflammatory mediators (Fig. 1A). We observed that i.v. and i.p. injections of ~5 × 10^5^ CFU of C. albicans led to its dissemination in multiple mouse organs (liver, spleen, and kidney), including the eye assessed at day 3 (D3) postinjection. However, the fungal burden was lower in the eyes than in other organs (Fig. 1B). Moreover, the i.v. route of inoculation resulted in a relatively higher C. albicans burden than the i.p. route. The presence of C. albicans in the eye coincided with a 20% to 30% reduction in ERG response as measured in terms of a-wave and b-wave amplitudes (Fig. 1C).

One of the hallmarks of endophthalmitis is the induction of intraocular inflammation. To this end, our data showed that C. albicans-infected eyes had increased levels of the inflammatory mediators interleukin 6 (IL-6), IL-1β, MIP2, and KC (Fig. 1D). Interestingly, the i.v. route of C. albicans administration induced a higher inflammatory response in the eye than that injected via i.p.

Since fungal infections are more prevalent in immunocompromised individuals, most experimental models have utilized immunosuppressive approaches to study the pathogenesis of fungal infections (25). However, the immunocompromised status tends to have increased mortality in animal models. In contrast, our data showed that intravenous injection of C. albicans, ranging from 10^4^ to 10^7^ CFU, did not cause mortality in immunocompetent mice observed for up to 10 days (see Fig. S1A in the supplemental material). The gross examination of kidneys showed abscess formation, but C. albicans burden was drastically reduced in all groups, with significant numbers of kidneys showing no viable C. albicans growth at 10 days postinfection (dpi) (Fig. S1B). In contrast, C. albicans was present in eyes of all experimental groups, even at 10 dpi (Fig. S1C).

Collectively, these results show that C. albicans can invade immunocompetent mice eyes during systemic infection and cause endophthalmitis. Moreover, the i.v. route of C. albicans administration results in a higher fungal burden and ocular pathology. Thus, we decided to choose the i.v. route of C. albicans injection for the rest of the study.

### C. albicans induces activation of inflammatory signaling in the mouse retina.

**FIG 2:**
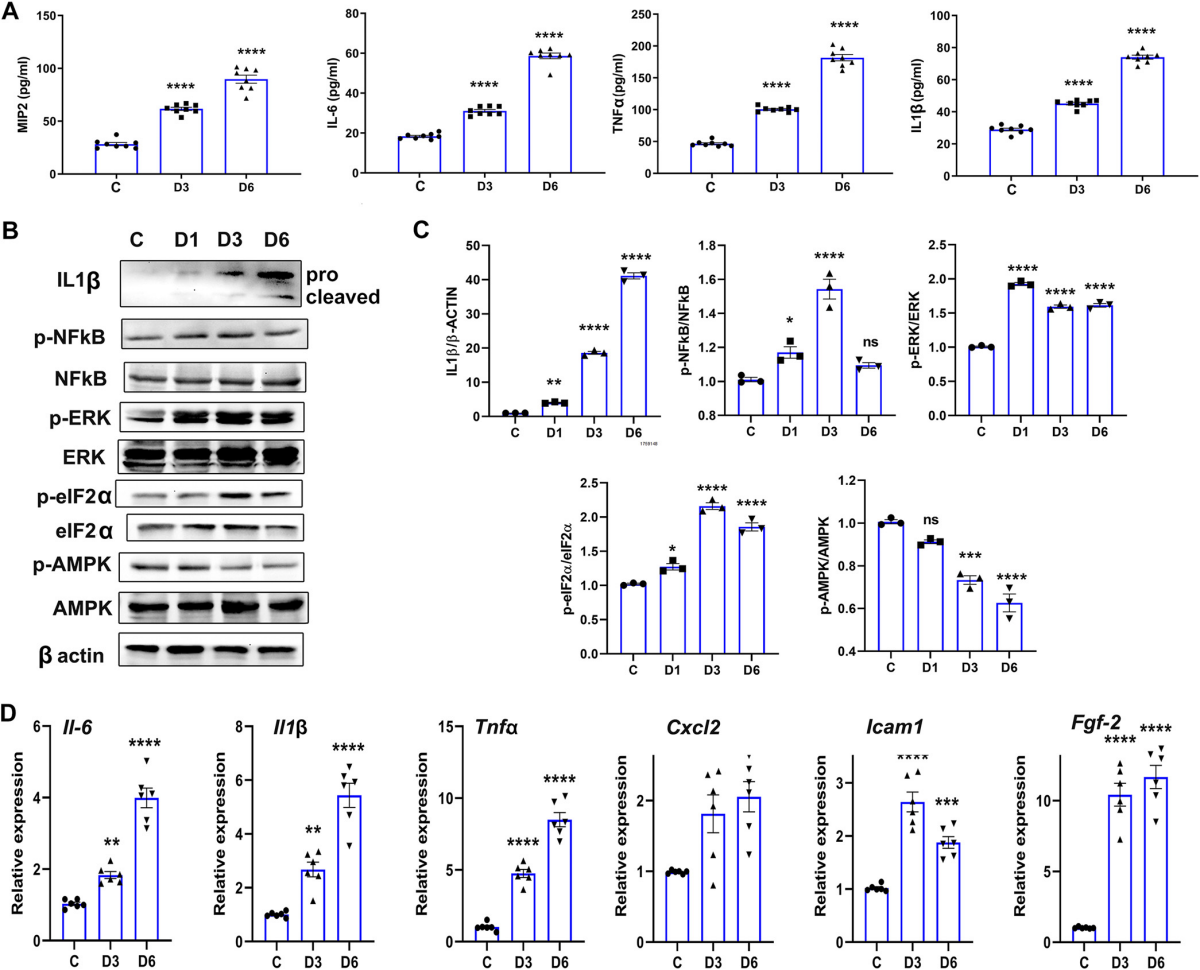
C. albicans-infected eyes exhibit an inflammatory response in the retina. (A) C57BL/6 mice (*n* = 8) were infected with C. albicans (~5 × 10^5^ CFU/mice) by the intravenous route, and eyes were enucleated on day 3 (D3) and day 6 (D6) postinfection for ELISA measurement of indicated inflammatory cytokines. In another experiment, retinal tissue was harvested from C. albicans-infected eyes at indicated days postinfection. (B to D) Western blotting (B) with densitometry (*n* = 3) (C) and qPCR analyses (*n* = 6) (D) were performed to assess the activation of inflammatory signaling and the expression of inflammatory response genes, respectively. Statistical analysis was performed using one-way ANOVA with Dunnett’s multiple-comparison test. *, *P* < 0.05; **, *P* < 0.01; ***, *P* < 0.001; ****, *P* < 0.0001; ns, nonsignificant.

Foregoing results show the invasion of C. albicans in mouse eyes on day 3. Next, we performed a time course study and observed that C. albicans-infected eyes had increased levels of inflammatory cytokines (MIP2, IL-6, tumor necrosis factor alpha [TNF-α], and IL-1β) on both day 3 and day 6 postinjection, with relatively higher levels at day 6 (Fig. 2A). As the enzyme-linked immunosorbent assay (ELISA) was performed on the whole-eye lysates, various ocular tissues may be contributing to the overall inflammatory response. However, during endogenous endophthalmitis, the main tissue affected is the retina and vitreous chamber. Thus, we assessed the effect of systemic C. albicans infection on mouse retinal tissue. We found that in comparison to uninfected control (C) eyes, the retinal tissue of C. albicans-infected mice exhibited activation of inflammatory signaling molecules (Fig. 2B). Our data showed a time-dependent increase in both pro-and active IL-1β levels. This coincided with an increase in levels of p-NF-κB, p-ERK, and p-eIF2α and the downregulation of p-AMPKα (Fig. 2C). Overall, significant changes were observed on day 3 and day 6 post-C. albicans injection. One of the outcomes of activation of NF-κB and mitogen-activated protein kinase (MAPK) signaling is the transcription and production of inflammatory mediators. Our quantitative PCR (qPCR) analysis revealed a time-dependent increase in the expression of inflammatory cytokines (e.g., IL-6, IL-1β, TNF-α, and CXCL2), cell activation markers (e.g., *Icam1*), and proangiogenesis growth factors (e.g., *Fgf-2*) transcripts in C. albicans-infected mouse retinal tissue (Fig. 2D). Together, these results indicate that systemic C. albicans infection evokes an inflammatory response in the retina.

### Systemic C. albicans infection impairs the outer blood-retinal barrier.

**FIG 3:**
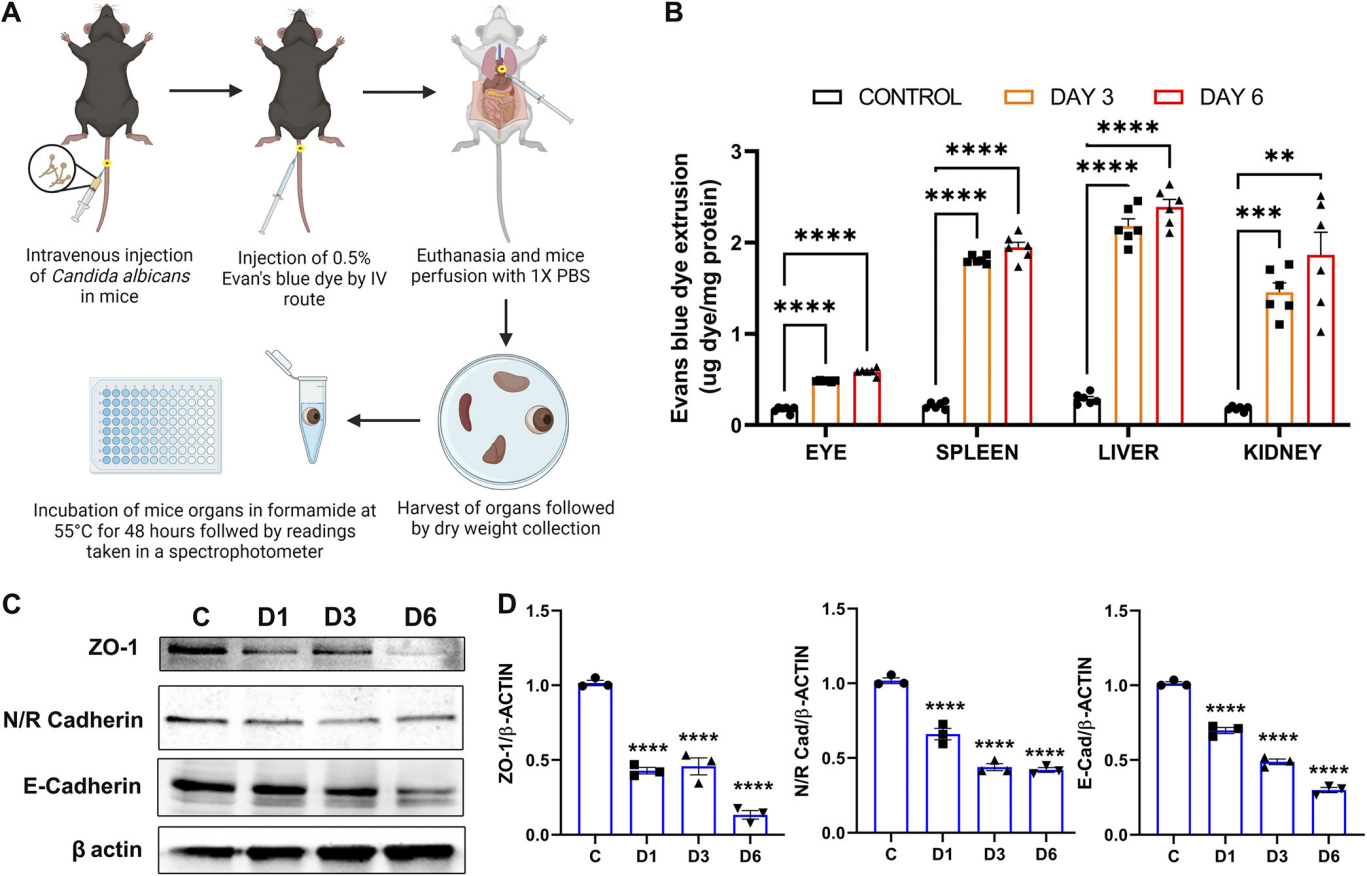
C. albicans infection breaches the blood-retinal barrier in the mouse eye. (A) C57BL/6 mice (*n* = 6) were infected with C. albicans (~5 × 10^5^ CFU/mouse) by the i.v. route. On day 3 (D3) or day 6 (D6), Evans blue dye was injected via the tail vein followed by euthanasia and cardiac perfusion. (B) The eyes and indicated organs were harvested for the estimation of the quantity of dye in the tissues and expressed as μg dye/mg protein of the tissue. (C) In another experiment, C. albicans-injected and PBS-injected (control [C]) retinal tissue was isolated on D1, D3, and D6 and used for immunoblotting. (D) Densitometry quantification (*n* = 3) was performed and expressed as fold change with respect to the control tissues. *, *P* < 0.05; **, *P* < 0.01; ***, *P* < 0.001; ****, *P* < 0.0001; one-way ANOVA with Dunnett’s multiple-comparison test.

Being an immune-privileged organ, the eye is protected from systemic pathogen circulation by the presence of the blood-retinal barrier (BRB). Our data showing the presence of C. albicans in the eye and the induction of inflammatory response in the mouse retina indicate the possibility of the breach of BRB. To test this, mice were injected with C. albicans via the i.v. route and on day 3 and day 6 postinjection, Evans blue dye (0.5%) was infused, and following the perfusion, the accumulation of the dye was assessed in various organs (Fig. 3A). Our data show that C. albicans-infected mice eyes had increased levels of Evans blue dye both on day 3 and day 6 (Fig. 3B). A similar accumulation of the dye was observed in other organs (e.g., spleen, liver, and kidney), but at relatively higher concentrations than in the eye.

Because several intracellular junction proteins maintain the BRB, we assessed the effect of C. albicans infection on these junction proteins by analyzing retinal tissue on day 3 and day 6 post-C. albicans injection. Our data showed a reduction in protein levels of ZO-1, N/R-cadherin, and E-cadherin in C. albicans-infected mouse retinal tissue by Western blot analysis (Fig. 3C and D). Overall, these results imply that C. albicans can gain access to the eye by downregulating cell junction proteins of the BRB.

**FIG 4:**
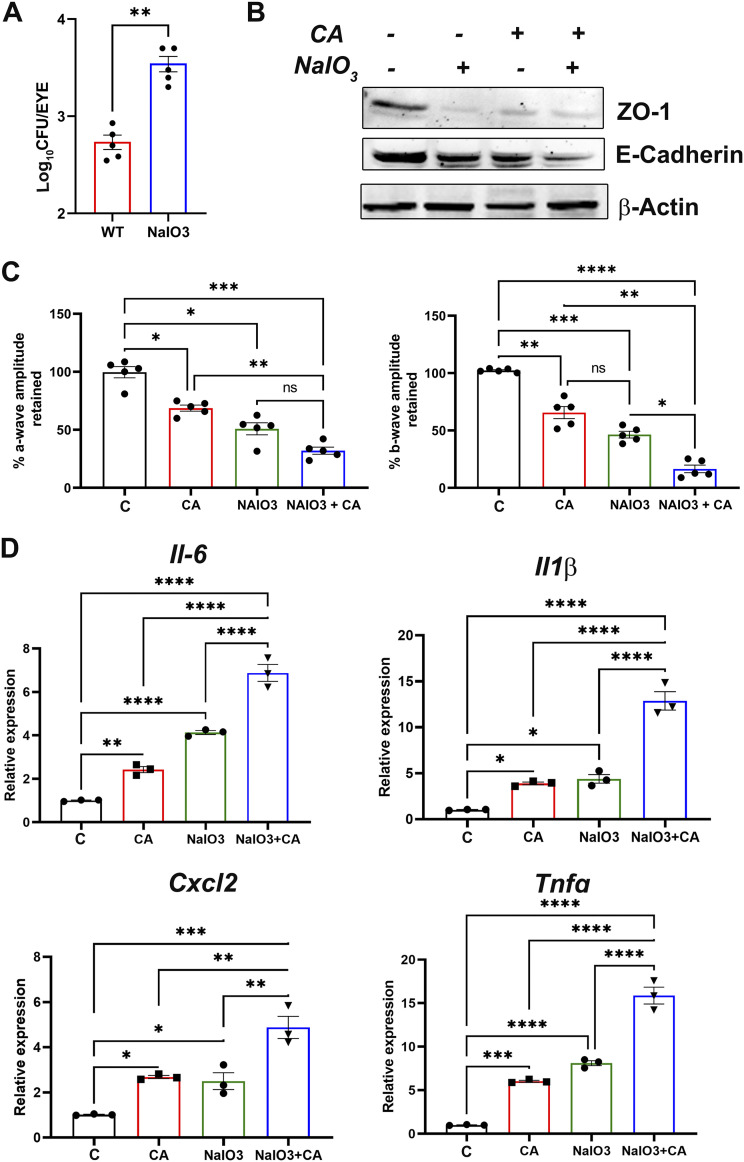
Disruption of outer BRB increases C. albicans invasion into the eye. C57/BL6 mice (*n* = 4 to 5) were pretreated with NaIO_3_ (50 mg/kg) followed by i.v. injection of C. albicans (~5 × 10^5^ CFU/mouse). The fungal burden in the mice eyes (A), intercellular junction protein levels (B), and the ERG response (C) were quantified on day 3 postinjection. (D) qPCR from the mice retinas (*n* = 3) was performed to assess the expression of inflammatory response genes. Statistical analysis was performed using unpaired *t* test (A) and one-way ANOVA with Tukey’s multiple-comparison test (B and C). ns, nonsignificant; *, *P* < 0.05; **, *P* < 0.01; ***, *P* < 0.001; ****, *P* < 0.0001.

BRB is maintained by retinal endothelial cells and retinal pigment epithelial (RPE) cells constituting the inner and outer BRB, respectively (23). Given the downregulation of ZO-1, abundantly expressed in RPE, we postulated the potential breach of outer BRB in our model. To determine the role of RPE cells in our model, mice were injected with sodium iodate (NaIO_3_) 24 h prior to systemic C. albicans infection. NaIO_3_ treatment compromises the outer BRB by inducing necroptosis in RPE cells (24, 26–28), resulting in decreased ERG response and enhanced inflammatory response (29, 30). Our data showed that intraocular fungal burden almost doubled in NaIO_3_-treated, C. albicans-injected mice (Fig. 4A). Moreover, NaIO_3_ treatment alone decreased levels of junction proteins, ZO-1, and E-cadherin in retinal tissue (Fig. 4B). The assessment of retina by ERG showed a significant decline in both a- and b-wave amplitudes in C. albicans-infected eyes, and reduction was higher in mice pretreated with NaIO_3_ (Fig. 4C). It should be noted that NaIO_3_ alone also reduced ERG response. Consistent with increased fungal burden, NaIO_3_-treated, C. albicans-injected mice exhibited higher levels of inflammatory mediators (IL-6, IL-1β, CXCL2, and TNF-α) than C. albicans or NaIO_3_ alone (Fig. 4D). Collectively, these findings demonstrate the ability of C. albicans to invade the eye by disrupting the outer BRB.

### C. albicans infection induces an innate immune response in cultured RPE cells.

**FIG 5:**
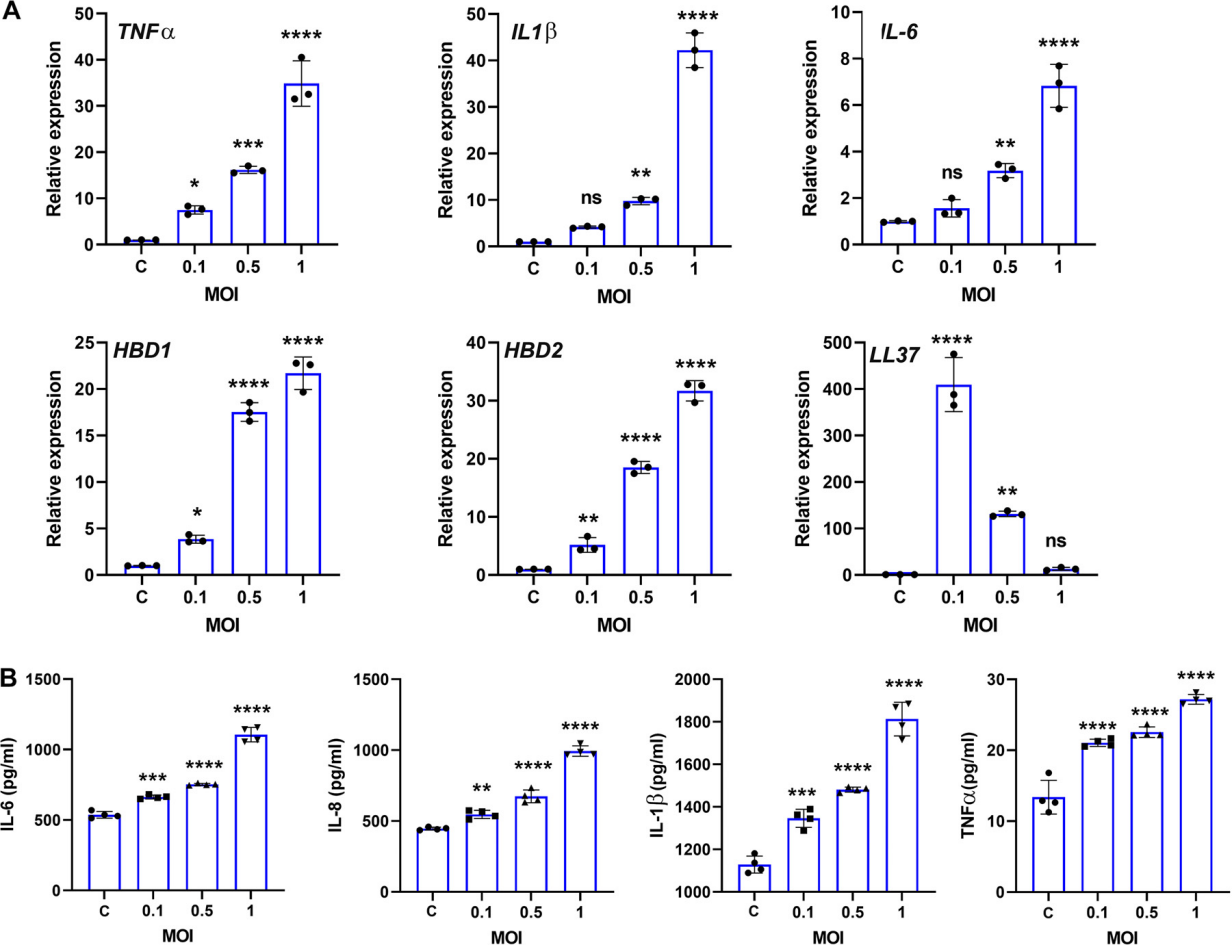
C. albicans triggers inflammatory and antimicrobial response in RPE cells. ARPE-19 cells (*n* = 3 to 4) were infected with C. albicans at different multiplicities of infection (MOIs) for 6 h. qPCR (A) and ELISA (B) were performed to check the expression and production of indicated inflammatory mediators. ns, nonsignificant; *, *P* < 0.05; **, *P* < 0.01; ***, *P* < 0.001; ****, *P* < 0.0001; one-way ANOVA with Dunnett’s multiple-comparison test.

Our *in vivo* data indicate the involvement of outer BRB in C. albicans endogenous endophthalmitis. To investigate the interaction of C. albicans with outer BRB, we adopted the *in vitro* model of cultured RPE cells. First, we performed a dose-response study by infecting ARPE-19 cells with C. albicans at various multiplicities of infection (MOIs) (0.1, 0.5, and 1) for 6 h. Our data showed dose-dependent expression of inflammatory mediators (TNF-α, IL-1β, and IL-6) and antimicrobial peptides (human β-defensin 1 [hBD1] and hBD2) except LL-37 in C. albicans-infected cells (Fig. 5A). The increased levels of inflammatory cytokines were also confirmed by ELISA (Fig. 5B). As C. albicans-infected RPE cells exhibited the highest inflammatory response at an MOI of 1, we selected this dose for all *in vitro* experiments.

**FIG 6:**
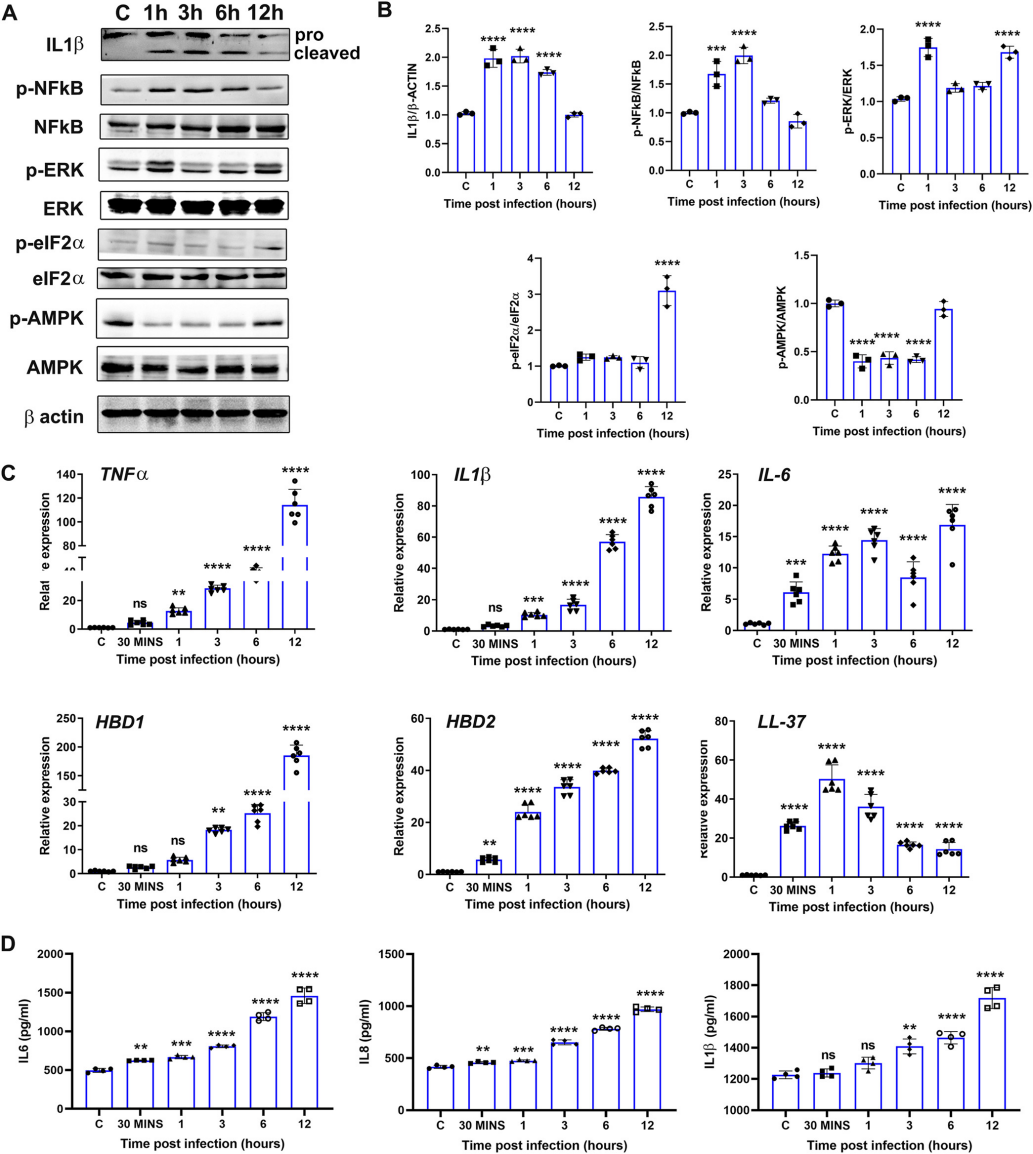
C. albicans-infected RPE cells exhibited activation of inflammatory signaling in a time-dependent manner. (A) ARPE-19 cells were infected with C. albicans at an MOI of 1 for different time points. Cells were used for immunoblotting (A), densitometry (B), and qPCR (*n* = 6) (C) detection of inflammatory cytokines, whereas culture supernatant (*n* = 4) was used for ELISA (D). ns, nonsignificant; *, *P* < 0.05; **, *P* < 0.01; ***, *P* < 0.001; ****, *P* < 0.0001; one-way ANOVA with Dunnett’s multiple-comparison test.

Next, we performed a time course study to assess the activation of inflammatory signaling in C. albicans-infected cells (Fig. 6A). To this end, our data showed a time-dependent increase in levels of both pro- and active IL-1β and phosphorylation of NF-κB, ERK, and eIF2α. The activation of ERK and AMPKα showed biphasic response with modulation at 1 h and 12 h postinfection (Fig. 6B). Coinciding with the activation of NF-κB signaling, C. albicans-infected RPE cells showed a time-dependent increase in the expression of inflammatory cytokines and antimicrobial peptides. However, the mRNA level of LL-37 increased early on but declined at later time points (Fig. 6C). The production of inflammatory cytokines (IL-6, IL-8, and IL-1β) at the protein levels also showed a time-dependent increase in culture media of C. albicans-infected cells (Fig. 6D). These results indicate that C. albicans infection triggers inflammatory and antimicrobial response in RPE cells.

### C. albicans infection increases transepithelial permeability in RPE cells.

**FIG 7:**
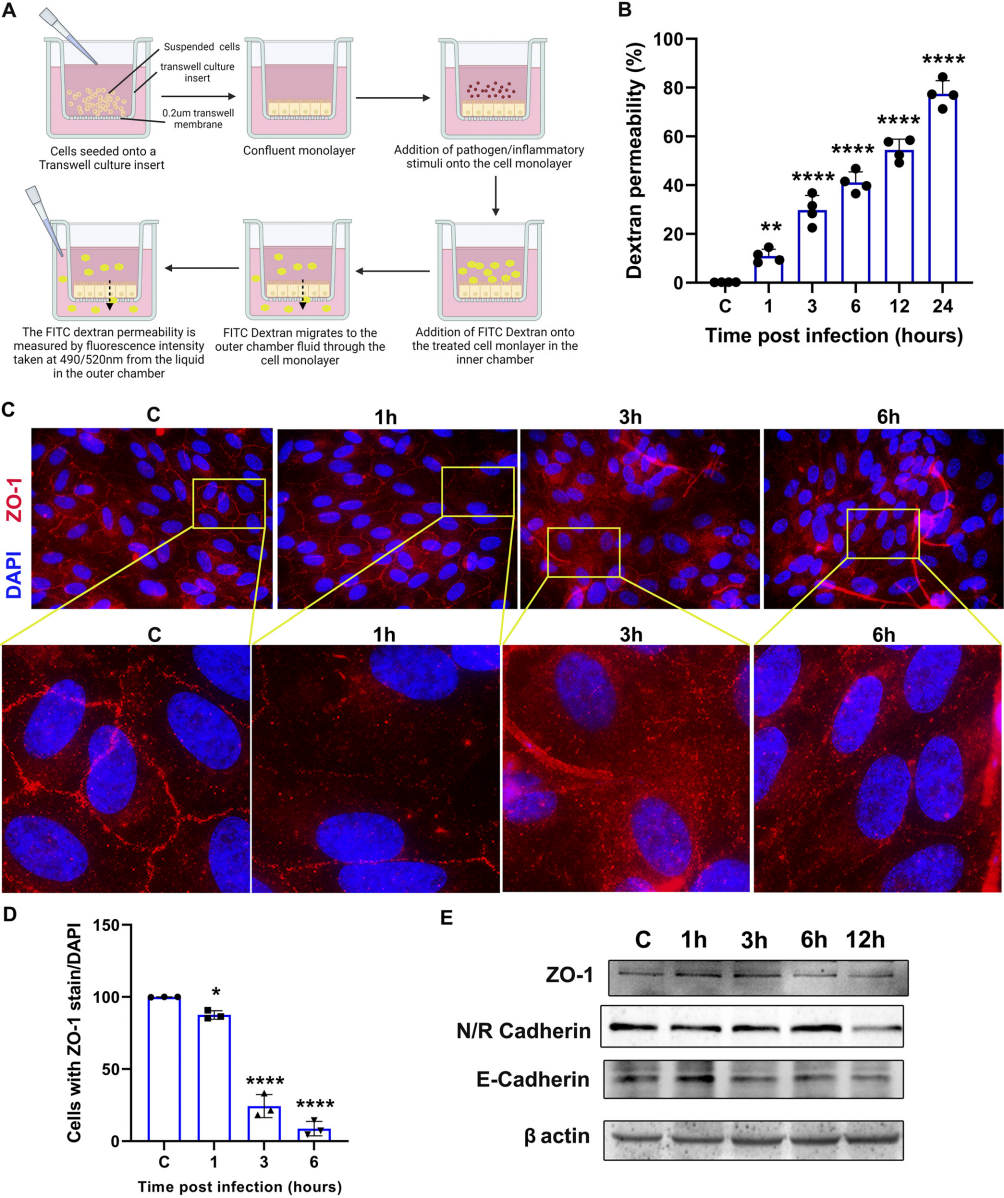
C. albicans infection decreases intracellular barrier integrity in RPE cells. (A) Schematic representation of the transwell FITC-dextran assay. (B) The dextran permeability of the C. albicans-infected ARPE-19 cells (*n* = 4) was expressed as percentage (%) compared to mock-infected control cells. (C) Cells infected with C. albicans were subjected to immunofluorescence staining to detect ZO-1 (red), and the cell nuclei were counterstained using DAPI (blue). The images were enlarged from specific regions indicated by yellow boxes. (D) The cells with complete ZO-1 staining at the cell periphery were counted with respect to the cell nuclei (*n* = 3). (E) In another experiment, C. albicans-infected cells were used for immunoblotting to check levels of indicated tight and adherens junction proteins. ns, nonsignificant; *, *P* < 0.05; **, *P* < 0.01; ***, *P* < 0.001; ****, *P* < 0.0001; one-way ANOVA with Dunnett’s multiple-comparison test.

Our *in vivo* data showed that systemic C. albicans infection resulted in endogenous endophthalmitis and C. albicans invasion into the mouse eye is increased by altering the RPE layer using NaIO_3_. To further investigate the role of outer BRB during C. albicans infection, we studied the effect of C. albicans infection on intracellular junction integrity using fluorescein isothiocyanate (FITC)-dextran transepithelial permeability assay. Briefly, ARPE-19 cells were seeded onto transwell culture inserts to reach confluence in 3 to 4 days and were infected with C. albicans at an MOI of 1. FITC-dextran was added to the upper chamber at different time points (ranging from 1 h to 24 h) post-C. albicans infection, and its concentrations were measured in the lower chamber (Fig. 7A). We found that C. albicans infection led to a significant increase in FITC-dextran permeability through the cell monolayer, and the response was time dependent, with higher levels in cells infected for 12 h (Fig. 7B). To determine whether the observed phenomenon was a result of the breakdown of intracellular junction proteins, an immunostaining assay was performed to visualize ZO-1 localization at the cellular junctions. The uninfected control ARPE-19 monolayer showed an intact cell-cell boundary stained for ZO-1, but its intensity was lost in C. albicans-infected cells at 3 hours postinfection (hpi) or 6 hpi (Fig. 7C) as quantified by cells with positive ZO-1 staining (Fig. 7D). The filamentous hyphae of C. albicans were also visible in the immunofluorescence assay (IFA) images. The decrease in the tight junction (ZO-1) and adherens junction (N/R-cadherin, and E-cadherin) proteins was confirmed by Western blot analysis (Fig. 7E). These results indicate that the increase in intracellular epithelial permeability was due to the loss of cell-cell junction proteins in the RPE cell monolayer upon C. albicans infection.

The foregoing results show that C. albicans infection impaired the outer BRB and evoked inflammatory response both *in vitro* and *in vivo*. Because both fungal cell wall (31) and secreted proteinases (32) of C. albicans contribute to virulence and pathogenesis, we sought to assess the effect of live and heat-killed C. albicans on RPE cells. First, we observed that in comparison to heat-killed C. albicans, live C. albicans infection caused significant cell death as measured by lactate dehydrogenase (LDH) release (Fig. S2A). Moreover, LDH levels increased in a time-dependent manner in live C. albicans*-*infected RPE cells (Fig. S2B). RPE cells exposed to heat-killed C. albicans exhibited significantly lower inflammatory and antimicrobial response than live C. albicans (Fig. S2C). The immunostaining showed disruption in membrane localization of ZO-1 in RPE cells challenged with live, but not the heat-killed, C. albicans (Fig. S2D). These results indicated the direct effects of C. albicans in inducing inflammatory response and causing loss of RPE barrier integrity.

## DISCUSSION

**FIG 8:**
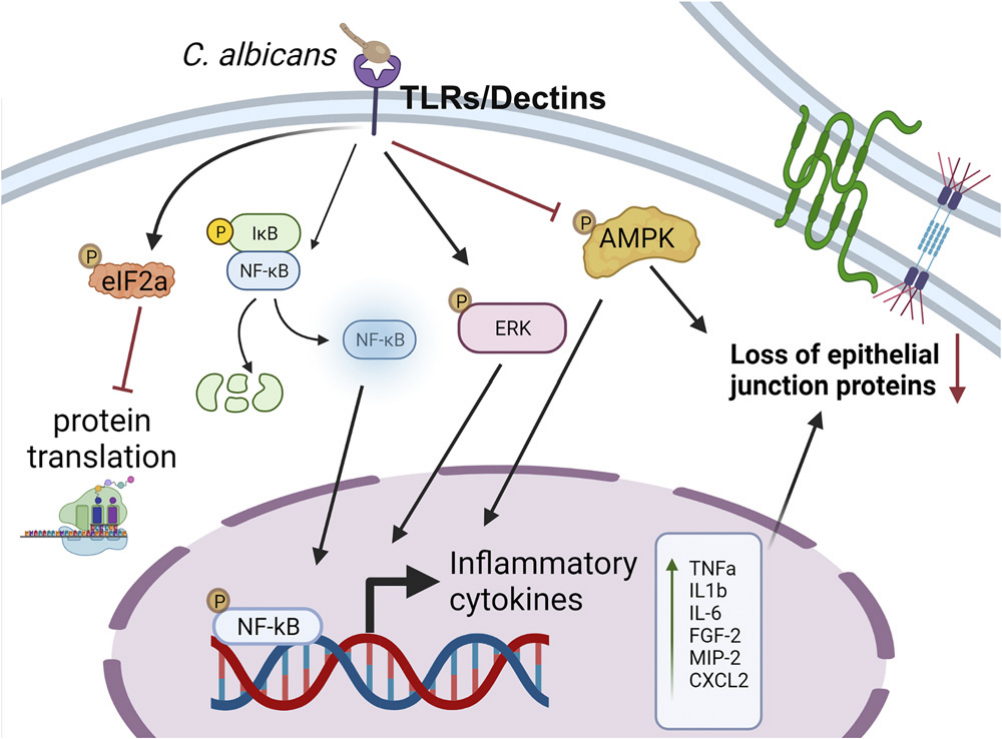
Schematics of *Candida* endogenous endophthalmitis pathogenesis. During systemic infection (i.e., candidemia), C. albicans in the choroidal blood vessels encounter RPE cells, which constitute the outer blood-retinal barrier (BRB). RPE cells recognize C. albicans via pathogen recognition receptors (e.g., TLRs or DECTINs) and trigger the cascade of inflammatory and stress signaling involving inflammation NF-κB, ERK, and eIF2α along with reduction in activated AMPK signaling. This results in the production of inflammatory cytokines/chemokines, which contribute toward increased cellular permeability. The figure was prepared using BioRender.

Endogenous endophthalmitis is a serious and devastating ocular infection. Due to their omnipresence, *Candida* species will continue to be the most common causative agents of endogenous fungal endophthalmitis (14–16, 33). The lack of information on host-pathogen interactions and the mechanisms of barrier property changes during ocular candidiasis have remained elusive, in part due to the lack of appropriate experimental models (4, 13, 22, 34–36). Therefore, we sought to determine the extent to which the BRB is compromised during C. albicans endophthalmitis by using our experimental fungal endogenous endophthalmitis murine model. Furthermore, we wanted to examine whether disruption of the cell-cell junctions comprising the ocular barrier contributed to this dysfunction. In this study, we demonstrated that systemic C. albicans injection in mice resulted in endogenous endophthalmitis. Our data show that C. albicans infection can impair the outer BRB by disrupting intracellular junction proteins in RPE cells, allowing C. albicans to invade the eye. Collectively, our study provides novel mechanistic insights into the pathogenesis of C. albicans endogenous endophthalmitis (Fig. 8).

Previous studies have used rabbit and mice models, with two routes of fungus administration, intravitreal and systemic (intravenous), to induce endophthalmitis (4, 13, 22, 34–36). Intravitreal injection directly introduces the pathogen inside the eye, causing vigorous inflammation, whereas the systemic infection in immunocompromised hosts results in early mortality without ocular involvement (4, 22, 37, 38). Consequently, these studies primarily focused on early and acute responses in fungal endophthalmitis. To mimic a clinical scenario, here, we used the intraperitoneal (i.p.) and intravenous (i.v.) routes for systemic administration of C. albicans in immunocompetent mice. Our dose-response and time course studies allowed us to assess innate immune response both at the early (day 1, day 3) and late (day 6) stages of infection. Moreover, because of the immunocompetent status of the mice, our data show that endogenous endophthalmitis can be induced without causing mortality, which is a major advantage of this model. Our survival study showed C. albicans was present in the eyes for up to 10 days, whereas it was cleared from most kidneys (primary target organ during candidemia). These results raise the possibility that like viruses (39), eyes might harbor fungal pathogens even though the patient recovered from systemic C. albicans infection. However, further investigation is needed to test this hypothesis.

Being a dimorphic fungus, C. albicans can exist in its yeast form, which aids in dissemination, and a filamentous form, where it can develop hyphae that can breach and invade tissues (40). During the initial phase of C. albicans infection, yeast cells adhere to the epithelium and/or endothelium. As it progresses, hyphal projections are produced, penetrating blood vessels, which ultimately allow it to gain circulatory access (11). Upon systemic candidiasis, the fungus encounters myeloid phagocytes, including neutrophils, inflammatory monocytes, tissue-resident macrophages, and dendritic cells (DCs), which mount strong innate defense (11). These effector cells act by phagocytosis and intracellular killing of C. albicans yeast cells by degranulation of antimicrobial molecules and neutrophil extracellular traps (NETs) formation to counteract extracellular fungal hyphae, production of both pro- and anti-inflammatory cytokines, and chemokines, AMPs, hydrolases, and nutritional immunity (41, 42).

The recognition of the C. albicans pathogen-associated molecular patterns (PAMPs) by host cell pattern recognition receptors (PRRs) like C-type lectin (CLR) superfamily, including DECTIN-1, -2, and -3, macrophage-inducible C-type lectin (Mincle), and Toll-like receptors (TLRs), triggers the development of an immune response (11, 43). Upon recognition, a signaling cascade involving NF-κB and mitogen-activated protein kinases (MAPKs) leads to the production of chemokines and cytokines; this allows innate immune sensing and control of C. albicans (44). Our data showing the activation of inflammatory signaling with the activation of NF-κB and ERK, along with induced expression of inflammatory genes in retinal tissues, points to the outcome of C. albicans invasion inside the eye, with a similar observation made in C. albicans-infected RPE cells. Therefore, the use of MAPK and ERK inhibitors would help in investigating their therapeutic role in preventing C. albicans endophthalmitis. In addition to inflammatory mediators, C. albicans-infected RPE cells exhibited induced expression of antimicrobial peptides (AMPs) such as beta-defensins and LL-37. These results indicate that RPE cells possess the ability to exert antimicrobial activity via secreted AMPs (11, 45–48). However, similar to our bacterial endophthalmitis study (49), the direct antifungal effects of these AMPs against C. albicans need to be investigated. We postulate that the protective response of RPE cells might be the reason for the few cases of ocular candidiasis in immunocompetent individuals. Interestingly, we observed that C. albicans induced the expression of fibroblast growth factor 2 (FGF-2), a known enhancer for the virulence of C. albicans, which promotes angiogenesis. Thus, blockage of FGF-2 has been proposed as a potential adjunct therapy to treat C. albicans infection (50), though further studies are warranted to investigate the role of FGF-2 in *Candida* endogenous endophthalmitis.

Among the various host molecules involved in regulating the inflammatory response against pathogens, our data revealed significant attenuation of AMPK signaling in the C. albicans-infected mouse retina and human RPE cells. AMPK is the master regulator of energy homeostasis and cell survival during stress (51, 52), and emerging evidence indicates that AMPK exerts anti-inflammatory effects by inhibiting NF-κB and other proinflammatory signaling (53). Therefore, upon C. albicans infection, reduced AMPK activity may augment the activation of the NF-κB cascade, leading to persistent inflammatory response in C. albicans endophthalmitis. Our prior studies demonstrate an essential role of AMPK in regulating the retinal innate immune response to ocular infection by modulating both residential (glial) and infiltrating immune cells. Therefore, pharmacological AMPK activators such as 5-aminoimidazole-4-carboxamide ribonucleotide (AICAR) and metformin can be potentially used to promote the inflammation resolution in C. albicans endogenous endophthalmitis (54–57).

To enter the eye and ultimately cause endophthalmitis, we hypothesize that C. albicans must disrupt the blood-retinal barrier (58) composed of retinal vascular endothelium and the retinal pigment epithelium. The retinal pigment epithelium is essential for maintaining the structural and functional integrity of the retina and the outer BRB. RPE cells also act as the first line of defense and express TLRs, complement components, and class I and II major histocompatibility complexes (MHC-I and MHC-II, respectively) and serve as antigen-presenting cells (59). During an ocular inflammation, the BRB functional state is altered, leading to increased permeability and recruitment of leukocytes in the retinal tissues. Therefore, to further understand the specific role of RPE cells in C. albicans invasion, NaIO_3_ treatment was utilized as a model for RPE degeneration (60). Our data showing increased C. albicans invasion and inflammatory response and reduced ERG function indicate that C. albicans causes endogenous endophthalmitis by directly affecting the retinal pigment epithelium. This coincides with similar findings reported in endogenous bacterial endophthalmitis (61). Together, these studies indicate the role of RPE cells in the pathogenesis of endogenous endophthalmitis.

The host response during fungal endophthalmitis leading to loss of BRB integrity is not well defined. Several cellular junction proteins maintain the BRB, and during bacterial infection and other ocular inflammation models (e.g., uveitis and glaucoma), increased inflammatory mediators (e.g., IL-6, MIP2, TNF-α, and IL-1β) can impair the barrier properties (24, 62–66). The involvement of proinflammatory cytokines and the loss of tight junction protein (ZO-1) have been implicated in endogenous bacterial endophthalmitis (24, 26, 61, 67). Our data using heat-killed C. albicans indicate that live C. albicans infection is required to disrupt the RPE layer’s cell-cell junction integrity, also demonstrated in the case of bacterial endophthalmitis (24). Since AMPK has been shown to play a role in regulating the barrier integrity, we postulate that reduced AMPK levels in our study might be contributing to the breach of outer BRB (57, 68). Hence, our study warrants further in-depth investigation of the role of AMPK in regulating the host response against C. albicans endophthalmitis to unravel a novel therapeutic approach to limit ocular candidiasis. Furthermore, apart from the host factors, Sap5p, one of the secreted proteases from C. albicans, can directly degrade E-cadherin (69). Therefore, deciphering the role of fungal proteases in disrupting the BRB integrity could provide novel insights into the pathogenesis of fungal endophthalmitis.

This is the first study to distinguish the host factors contributing to inflammation and permeability of the blood-retinal barrier and establish variations in the RPE tight and adherens junctions during fungal endophthalmitis. Our novel findings on the involvement of inflammatory mediators and AMPK in C. albicans ocular infection and BRB permeability provide a platform for further studies to comprehend the mechanism of BRB dysfunction and endophthalmitis. In summary, our study provides novel mechanistic insights into both the pathogenesis of C. albicans endophthalmitis and the breach of the BRB in fungal endophthalmitis. Moreover, the experimental models developed in this study can be utilized to identify both host and pathogenic factors contributing to the development of endogenous endophthalmitis and also identify therapeutic drugs intended to recover the stability of BRB.

## MATERIALS AND METHODS

### Mice and ethics statement.

C57BL/6J mice (both male and female, 6 to 8 weeks of age) were procured from the Jackson Laboratory (Bar Harbor, ME) and were maintained in the Division of Laboratory Animal Resources (DLAR) facility of the Kresge Eye Institute, Wayne State University. The mice were kept on an alternate 12-h light/dark cycle and fed LabDiet rodent chow (LabDiet, PicoLab, St. Louis, MO, USA). All procedures involving mice were performed as per the Guide for the Care and Use of Laboratory Animals, the Wayne State University IACUC, and the Association for Research in Vision and Ophthalmology Statement for the Use of Animals in Ophthalmic and Vision Research.

### C. albicans endogenous endophthalmitis model.

Candida albicans (strain sc-5314; ATCC) was maintained on yeast extract-peptone-dextrose (YPD) agar plates (Sigma-Aldrich). Prior to mouse inoculation, C. albicans was grown at 30°C in YPD broth for 18 h to 24 h, and the infectious dose was adjusted by resuspending in sterile phosphate-buffered saline (PBS) at approximately 2.5 × 10^6^ to 3.5 × 10^6^ CFU/mL. Mice were injected with 200 μL (i.e., 5 × 10^5^ CFU/mouse) via intraperitoneal or intravenous (lateral tail vein) routes. The eyes were enucleated on days 1, 3, and 6 postinfections for various assays described in the following sections. C. albicans was heat killed at 95°C for 10 min followed by plating for confirmation of the process of fungal inactivation.

### *In vitro* outer blood-retinal barrier model.

Human retinal pigment epithelial cells (ARPE-19 cell line; ATCC CRL-2302) were maintained in Dulbecco’s modified Eagle medium (DMEM)–F-12 culture medium (Invitrogen) supplemented with 10% heat-inactivated fetal bovine serum (FBS; Invitrogen) and 1% penicillin-streptomycin solution (Corning) (70). The cells were grown in a humidified chamber (Thermo Fisher Scientific) at 37°C and 5% CO_2_. Cells were challenged with C. albicans sc-5314 at various MOIs or time points to perform dose-response and time course studies.

### Fungal burden estimation.

Mice were euthanized at 1, 3, and 6 days post-C. albicans injection, followed by extensive intracardiac perfusion using sterile PBS to remove blood from the circulation. The internal organs (eyes, kidney, spleen, and liver) were aseptically harvested and weighed, and tissue homogenates were prepared using the bead-crushing method. The homogenates were serially diluted in sterile PBS and plated on YPD agar plates (4). The fungal burden was enumerated by CFU count after overnight incubation at 30°C. The fungal burden in infected eyes is expressed as log_10_ CFU/eye, whereas log_10_ CFU/g tissue was used for calculation in other organs.

### Evans blue permeability assay.

The breach of the blood-retinal barrier was quantified after intravenous (i.v.) injection of C. albicans using the modified albumin-Evans blue dye extrusion assay as previously described (61). Briefly, C. albicans or mock-injected mice were anesthetized with ketamine and xylazine, and albumin-Evans blue dye (30 mg/mL; Sigma-Aldrich) was intravenously administered 2 h prior to euthanasia. Intracardiac perfusion was performed through the left ventricle with saline to remove intravascular albumin-Evans blue. Mice were then euthanized, and organs, including the eye, were harvested, weighed, and suspended in formamide to extract the dye at 55°C for 48 h. The concentration of the dye was measured by spectrophotometer (620 nm absorbance), and values were calculated using a standard curve of albumin-Evans blue dye normalized to the total protein in the sample. The results (mean ± standard error of the mean [SEM]) were expressed as micrograms of dye per milligram of tissue protein content.

### ERG analysis.

Scotopic electroretinography (ERG) was performed to evaluate retinal visual function in control and C. albicans*-*injected mouse eyes using the Celeris ERG system (Diagnosys LLC, Lowell, MA, USA) as described previously (71). Data were analyzed with respect to control eyes and expressed as percentage of wave amplitude retained.

### LDH release assay.

The cellular toxicity was performed using LDH release kit as per the manufacturer’s instructions (Abcam, CA, USA). Briefly, 10 μL of RPE cell culture supernatant was mixed with kit reagents and incubated for 30 min at room temperature. The LDH activity was quantified using BioTek plate reader at 535/587 nm. The LDH release was normalized compared to the uninfected control and expressed as LDH release (percentage of the age of control).

### Real-time PCR.

Total RNA was extracted from the retinal tissue or cultured ARPE-19 cells using TRIzol reagent as per the manufacturer’s instructions (Invitrogen, Carlsbad, CA, USA). cDNA was synthesized from 1.0 μg of total RNA using a Maxima first-strand cDNA synthesis kit (Thermo Fisher Scientific, Rockford, IL, USA) as per the manufacturer’s instructions. The quantitative PCR (qPCR) was performed to assess the expression of genes regulating inflammatory cytokines/chemokines, antimicrobial peptides, and intracellular junction proteins using the StepOnePlus PCR instrument (Applied Biosystems, Grand Island, NY, USA). Gene expression was analyzed by the threshold cycle (ΔΔ*C_T_*) method and normalized with *Gapdh*, an endogenous housekeeping reference gene.

### ELISA.

The production of inflammatory cytokines and chemokines was determined using commercially available enzyme-linked immunosorbent assay (ELISA) kits. Twenty micrograms of total protein from whole-eye lysates or 100 μL of the cell culture supernatant were used for individual assays. All ELISAs (TNF-α, IL-1β, IL-6, KC, and CXCL2/MIP2) were performed as per the manufacturer's instructions (R&D Systems, Minneapolis, MN, USA). The data were presented as mean cytokine/chemokine concentrations (picogram per milligram of eye lysates) mean ± SEM for mice and picogram per milliliter (mean ± SD) for cell culture supernatant.

### Western blot analysis.

For Western blot analyses, hRPE cells were lysed in radioimmunoprecipitation assay (RIPA) lysis buffer (Thermo Scientific), whereas mouse retinal tissue was lysed by sonication in PBS, and the protein concentrations were estimated using the bicinchoninic acid (BCA) assay kit (Thermo Scientific) as per the manufacturer’s instructions. Denatured proteins were resolved on 10% sodium dodecyl sulfate-polyacrylamide gel electrophoresis (SDS-PAGE) and transferred onto 0.45-μm nitrocellulose membranes (Bio-Rad, Hercules, CA). The membrane was blocked using 5% nonfat skim milk for whole proteins or in 5% bovine serum albumin (BSA; Sigma-Aldrich) for phosphoproteins, followed by a wash with 1× Tris-glycine buffer with Tween 20 (TBST). Blots were incubated with the primary antibody diluted using 5% BSA in TBST overnight at 4°C with shaking. After secondary antibody incubation, protein bands were visualized using SuperSignal West Femto chemiluminescent substrate (Thermo Scientific, Rockford, IL). For semiquantitative analyses, intensities of protein bands were measured using ImageJ software (NIH, USA).

### FITC-dextran permeability assay.

The *in vitro* transwell FITC-dextran permeability assay was performed as described previously (72, 73). Briefly, ARPE-19 cells were seeded onto fibronectin (10 μg cm^−2^; Sigma-Aldrich)-coated transwell tissue culture inserts (6.5 mm diameter, 0.4 μm pore size; Corning Costar; catalog no. CLS3470) placed on a 24-well plate. The cell density was 5 × 10^4^ cells per insert, and they were cultured in complete DMEM–F-12 cell growth medium in both upper and lower chambers. Upon confluence (48 h postseeding), cells were infected with C. albicans at an MOI of 1 or mock infected. FITC-dextran, 40, 000 molecular weight (MW; catalog no. FD40S; Sigma-Aldrich), constituted 150 μL of 1 mg/mL in 1× Hanks’ balanced salt solution (HBSS) was added onto the upper chamber, and 600 μL of 1× HBSS was added to the lower chamber. After 1 h of incubation at 37°C, the amount of FITC-dextran permeated into the lower chamber was quantified using absorbance measurement with 490 nm excitation and 530 nm emission (Synergy LX multimode reader). Dextran permeability was expressed as a percentage increase over the basal permeability observed in the mock-infected monolayer at the respective time points. The data are presented as mean ± SD.

### Immunofluorescence staining.

ARPE-19 cells were cultured in four-well chamber slides (Nunc Lab-Tek) to confluence and allowed to form mature epithelial structures by growing in low-serum media (5% FBS) for 3 days. The confluent monolayer was infected with C. albicans at an MOI of 1 for different time points. The cells were fixed using 4% paraformaldehyde in 1× PBS overnight at 4°C. The cells were washed thrice with 1× PBS and then blocked using 1% BSA in 1× PBS for 1 h at room temperature. The cells were then incubated with rabbit anti-ZO1 IgG (1:100) in the dilution buffer in a humidified chamber overnight at 4°C followed by three washes with 1× PBS. The cells were then incubated with anti-rabbit Alexa Fluor 594 (Invitrogen) for 1 h at room temperature in a humidified chamber. The cells were washed thrice with 1× PBS and mounted in Vectashield antifade mounting medium (Vector Laboratories). The cells were visualized using the Keyence fluorescence microscope at ×600 magnification. The cells with intact peripheral ZO-1 staining were counted against the DAPI (4′,6-diamidino-2-phenylindole)-stained cells to detect the nuclei using ImageJ software and represented as cells with ZO-1 staining/DAPI.

### Statistical analysis.

Statistical analysis was performed using GraphPad Prism 9 software, and the data are presented as mean ± SEM for animal studies or mean ± standard deviation (SD) for *in vitro* studies. A *P* value of <0.05 was considered statistically significant. All experiments were performed at least three times unless mentioned otherwise.

### Data availability.

The data that support the findings of this study are available from the corresponding author upon reasonable request.
