# Supplementary material for: Systemic Candida albicans Infection in Mice Causes Endogenous Endophthalmitis via Breaching the Outer Blood-Retinal Barrier
Source: Microbiol Spectr. 2022 Aug 1;10(4):e01658-22. doi: 10.1128/spectrum.01658-22 (PMC9431129; doi:10.1128/spectrum.01658-22)
Supplement: Supplemental file 1 — Fig. S1 and S2. Download spectrum.01658-22-s0001.pdf, PDF file, 0.3 MB [file spectrum.01658-22-s0001.pdf]

Systemic *Candida albicans* infection in mice causes endogenous endophthalmitis via breaching the outer blood-retinal barrier

Authors: Sneha Singh[1], Sukhvinder Singh[1], Ashok Kumar[1,2]

Author Affiliations:

[1]Department of Ophthalmology, Visual and Anatomical Sciences/ Kresge Eye Institute, Wayne State University School of Medicine, Detroit, MI, USA.

[2]Department of Biochemistry, Microbiology, and Immunology, Wayne State University School of Medicine, Detroit, MI, USA.

Running Title: *Candida albicans* endophthalmitis and blood-retinal barrier

***Corresponding Author:** Ashok Kumar, Ph.D.

Department of Ophthalmology, Visual and Anatomical Sciences

Wayne State University School of Medicine

4717 St. Antoine, Detroit, MI 48201

Tel: (313) 577-6213

E-mail: akuma@med.wayne.edu

Supplementary Data:

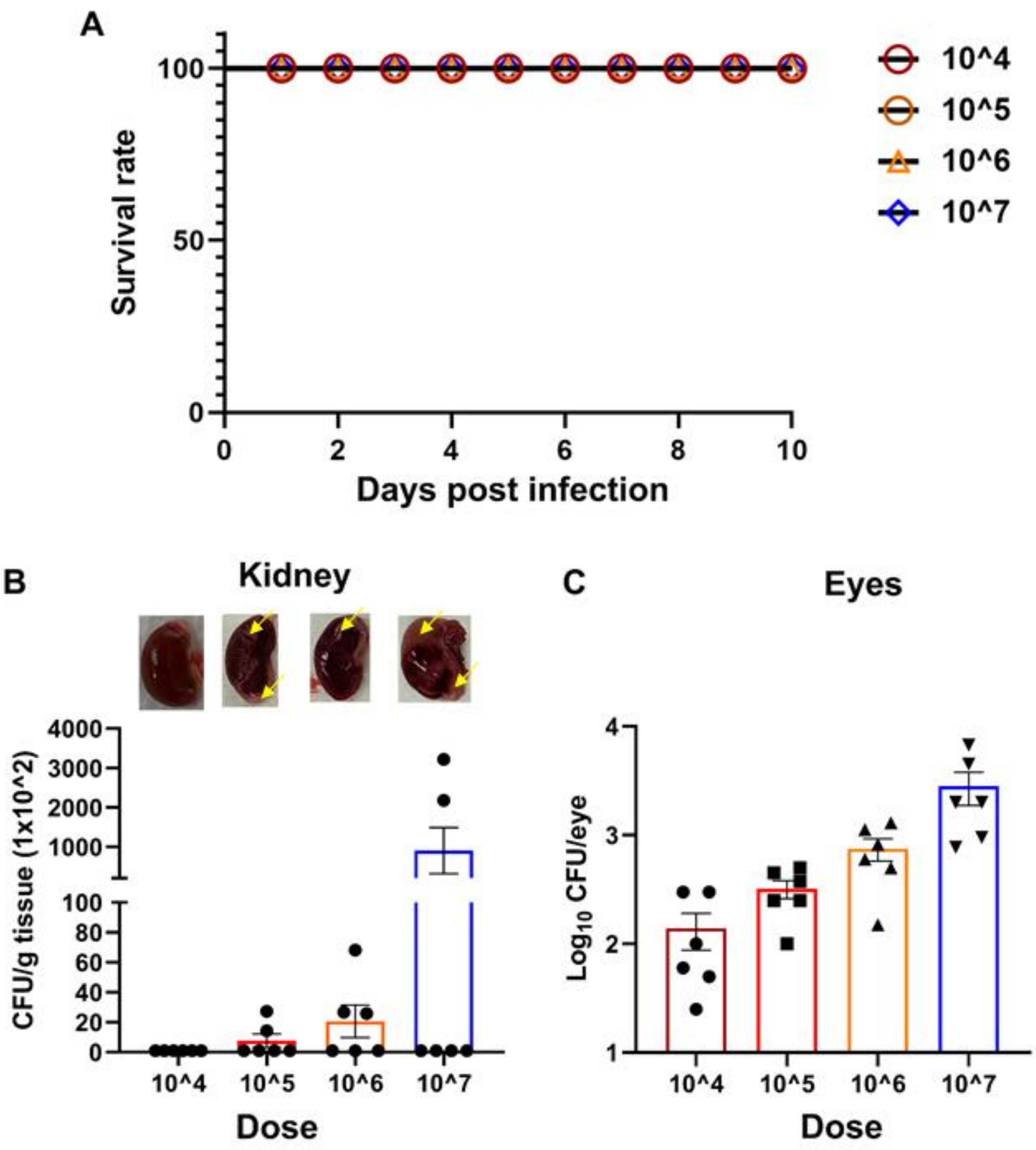

Supplementary (S) Figure 1: Survival curve and fungal burden in *C. albicans* infected B6 mice. The B6 mice were injected with increasing doses of *CA* (10^4, 10^5, 10^6, and 10^7 CFU) intravenously followed by daily observation up to 10 days. The Kaplan-Meier survival graph was plotted (**A**). The fungal burden in kidneys as well as in eyes were estimated by standard plate count and plotted as $\log_{10}$ CFU/g tissue for kidneys (**B**) and $\log_{10}$ CFU/eye for intraocular (**C**). The arrows indicate the abscesses.

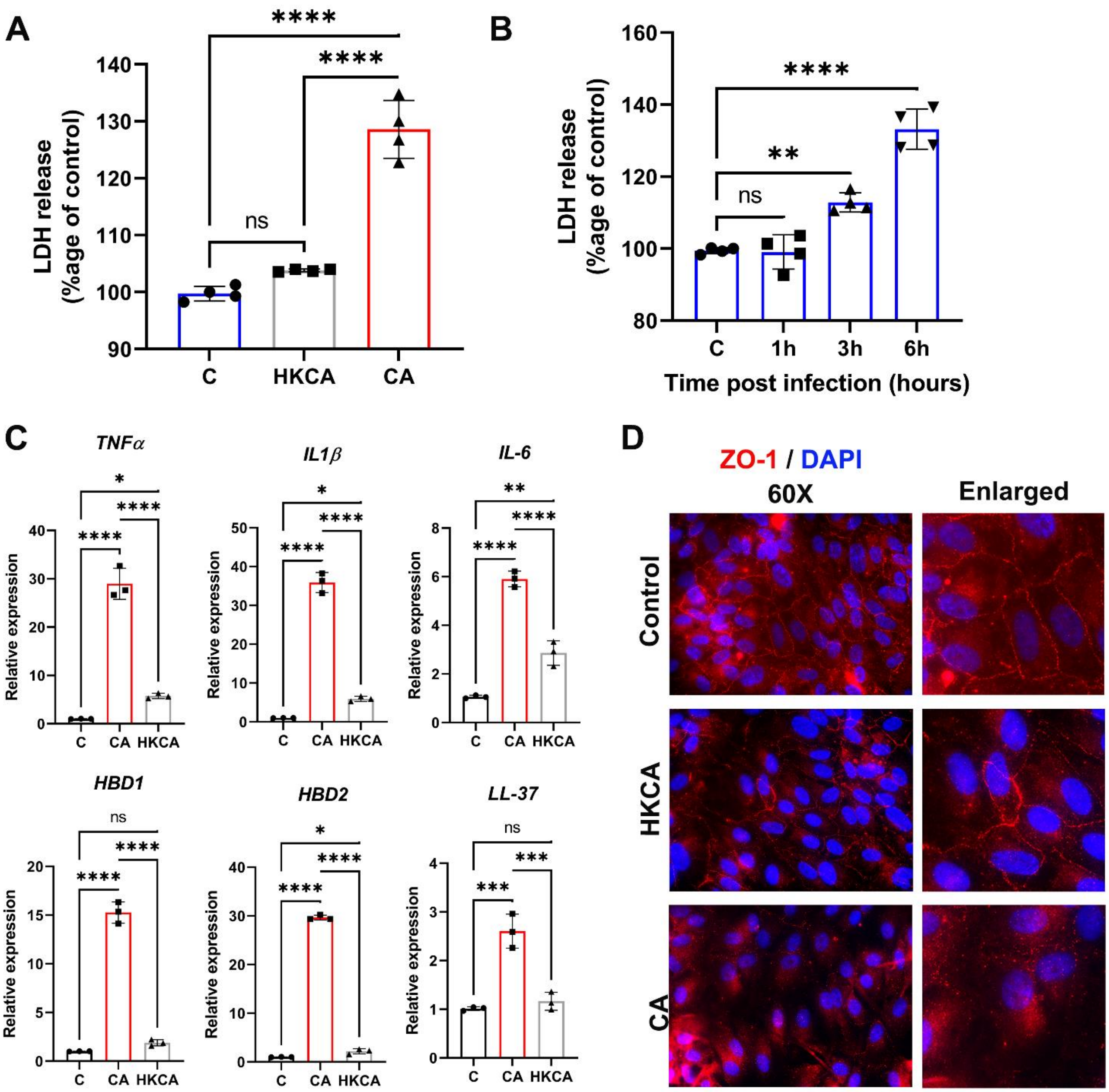

Supplementary (S) Figure 2: Live *C. albicans* infection causes loss of intracellular barrier integrity and increased inflammation in RPE cells. ARPE-19 cells were infected with live (*CA*) or heat-killed *CA* (HKCA) at MOI 1 for different time points. The cell culture supernatant (n=4) was used for LDH release quantification (**A & B**), and qPCR analysis (n=3) of inflammatory cytokines (**C**). Cells infected with *CA* and HKCA were subjected to immunofluorescence staining (**D**) to detect ZO-1 (red) and the cell nuclei were counterstained using DAPI (blue). The images were enlarged to highlight specific regions.
